# Highly Sensitive Gas and Ethanol Vapor Sensors Based on Carbon Heterostructures for Room Temperature Detection

**DOI:** 10.1021/acsami.4c21591

**Published:** 2025-02-21

**Authors:** Michal Kočí, Pawel S. Wrobel, Marcin Godzierz, Ondrej Szabó, Sławomira Pusz, Štěpán Potocký, Miroslav Husák, Alexander Kromka

**Affiliations:** † Department of Semiconductors, Institute of Physics of the Czech Academy of Sciences, Cukrovarnická 10/112, Prague 6 162 00, Czech Republic; ‡ Department of Microelectronics, Faculty of Electrical Engineering, Czech Technical University in Prague, Technická 2, Prague 6 166 27, Czech Republic; § Centre of Polymer and Carbon Materials of the Polish Academy of Sciences, ul. M. Curie Skłodowskiej 34, Zabrze 41-819, Poland; ∥ Łukasiewicz Research Network−PORT Polish Center for Technology Development, ul. Stabłowicka 147, 54-066 Wrocław, Poland

**Keywords:** graphene oxide, reduced graphene oxide, thiol-functionalized graphene oxide, hydrogen-terminated nanocrystalline diamond, ethanol vapor detection, gas sensor

## Abstract

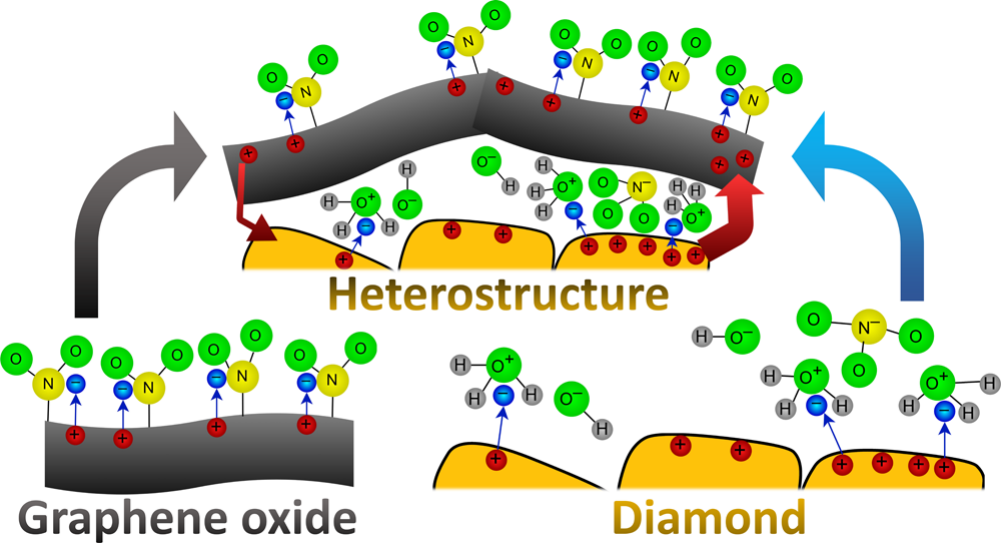

Graphene oxides (GOs) and hydrogen-terminated nanocrystalline diamonds (H-NCD) have attracted considerable attention due to their unique electronic structure and extraordinary physical and chemical properties in various applications, including gas sensing. Currently, there is a significant focus on air quality and the presence of pollutants (NH_3_, NO_2_, etc.), as well as volatile organic compounds (VOC) such as ethanol vapor from industry. This study examines the synthesis of GO, reduced graphene oxide (rGO), thiol-functionalized graphene oxide (SH-GO), and H-NCD thin films and their combination in heterostructures. The materials were analyzed for their ability to detect NO_2_, NH_3_, and ethanol vapor at room temperature (22 °C). Among the tested materials, the SH-GO/H-NCD heterostructure exhibited the highest sensitivity, with approximately 630% for ethanol vapor, 41% for NH_3_ and −19% for NO_2_. The SH-GO/H-NCD heterostructure also demonstrated reasonable response (272 s) and recovery (34 s) times. Cross-selectivity measurements revealed that the heterostructure’s response to ethanol vapor at 100 ppm remained dominant and was minimally affected by the presence of NH_3_ (100 ppm) or CO_2_ (100 ppm). The response variations were −1.3% for NO_2_ and 2.4% for NH_3_, respectively. These findings suggest that this heterostructure has the potential to be used as an active layer in low-temperature gas sensors. Furthermore, this research proposes a primary mechanism that explains the enhanced sensor response of the heterostructure compared with bare GOs and H-NCD layers.

## 1 Introduction

The quality of life on Earth is strongly related to the cleanliness of the air. The modern world is heavily reliant on industry, which is one of the leading air pollutants in the 21st century. Typically, harsh inorganic compounds like ammonia (NH_3_), carbon monoxide (CO), carbon dioxide (CO_2_), sulfur oxides (SO_*x*_), or nitrogen oxides (NO_*x*_) are released into the atmosphere.^1,2^ Moreover, industries associated with polymers, paints, aerosols, oil processing, and other industries heavily rely on organic solvents. This reliance leads to pollution in the form of VOC. A representative of VOCs is ethanol (C_2_H_5_OH), one of the industry’s most commonly used solvents.^3,4^ In addition, ethanol (alcohol) is a psychoactive substance related to up to one-quarter of fatal traffic accidents, according to the European Road Safety Observatory (ERSO) report for the European Commission.^5^ Therefore, monitoring its concentration in the air with a stable response, high sensitivity (below 10 ppm),^2,6^ and good reproducibility, ideally operating at room temperature (around 25 °C), is still challenging. According to the WHO (World Health Organization),^6^ the maximum concentration at which the organism is unaffected is 8.58 ppm for CO, 1.3 ppm for NO_2_, and 2.46 ppb for benzene. The typical concentration of ethanol vapor that will affect the body is in the range of 250–1000 ppm.^7^ It is, therefore, essential to use sensors with detection limits below this level.

For this reason, high demands are made on developing new types of sensors, especially with lower production costs and improved sensitivity to gaseous media.^2^ Nowadays, two materials for gas sensors can be distinguished–highly resistive ceramic structures^8−13^ or conductive carbon-based structures.^14−17^ Typically, the sensing system is designed for a specific application, such as the detection of ammonia,^15,18,19^ acetone,^11,20,21^ ethanol,^3,4,21−23^ methanol,^3,9,24^ hydrazine,^25^ or NO_2_.^14,15,17^ Therefore, the fabricated gas sensors should be very selective. Additionally, depending on the type of conductivity of the carbon-based sensors, different gas mixtures will affect the resulting conductivity through different chemical interactions between the sensor surface and the gases, causing the sensor resistance to increase or decrease based on the nature of the gas.^1,2,16,17,26−30^

To improve the performance of carbon-based sensors, one can enhance it by functionalizing the carbon materials through oxidation,^15,17^ chemical modifications,^31^ or by increasing the specific surface area.^32^ Among carbon-based gas sensors, mostly graphene and its derivatives, e.g., rGO^17,21,23^ and carbon nanotubes (CNTs),^15,16^ are suitable candidates for gas sensing applications. Gas sensors based on rGO and CNT structures show measurable responses (about 1.2%) even to deficient concentrations (5 ppm) of ethanol vapor.^16,17^ Another way to improve the performance of carbon-based gas sensors, as reported in the literature, is the fabrication of hybrid or heterostructures, which consists of carbon nanostructures with polymers,^18,19,33^ ceramic nanoparticles^20,24,34^ or a combination of both.^35^ For example, the results presented by Shooshtari et al.^16^ for CNT and CNT/TiO_2_ systems, where CNT/TiO_2_ heterostructure shows 3 and 3.5 times higher sensitivity to ethanol and acetone vapor at low concentrations (5 ppm) than pure materials. Gavgani et al.^18^ have demonstrated that fabricated S and N codoped graphene quantum dots/PANI hybrid system shows three times higher sensitivity to ammonia than pure PANI.

This paper presents the sensing performance of hybrid carbon-based conductive gas sensors operating at room temperature. The gas sensors were prepared with various detecting layers H-NCD,^14,29^ GO,^31^ rGO,^31^ and SH-GO.^31^ Combining these materials into one active layer enhances the gas sensing parameters for ethanol vapor, ammonia, and nitrogen dioxide compared to stand-alone sensors (2.5% to 100 ppm of NH_3_ for H-NCD^14^ or 40% to 100 ppm ethanol for SH-PRGO^31^). It was found that the SH-GO/H-NCD heterostructure shows the highest sensitivity to detect ethanol vapor, even at low concentrations (10 ppm). Additionally, such heterostructure also significantly improves the sensitivity toward NH_3_ and NO_2_.

## 2 Materials and Methods

### 2.1 Sensor Preparation

The active materials of the gas sensors were prepared on a glass substrate with an interdigital (IDT) structure. The IDT structure from Micrux Technologies (with 200 nm Ti/Au electrodes) consists of 90 pairs of 10 μm width electrodes with a 10 μm gap between them. The diameter of the IDT structure is 3.5 mm. The dimensions of the whole substrate are 10 mm × 6 mm. The substrates were subjected to an ultrasonic cleaning process in acetone, followed by a cleaning in isopropyl alcohol and then deionized water for 10 min.

#### 2.1.1 H-NCD Layers

The preparation of NCD layers was previously described in our works.^14,29,36^ The layers were prepared by microwave-plasma-enhanced chemical vapor deposition (MW-PECVD) in four deposition steps.

First, the diamond nucleation layer was prepared by substrate treatment in a water-based nanodiamond powder suspension (NanoAmando, with nominal particle size 5 nm) in an ultrasound bath for 40 min.

To reduce induced thermal stress, due to different thermal expansion coefficients of the glass substrate and NCD layer, an adhesive interlayer (also made of diamond) was deposited at a low temperature (less than 400 °C) in a MW-PECVD system (Roth and Rau AK400) with linear antennas.^36^ The process parameters were as follows: MW power 1700 W and working pressure 0.15 mbar of a 150 sccm H_2_, 5 sccm CH_4_, and 20 sccm CO_2_ gas mixture. The deposition time was 20 h. During deposition, the temperature did not exceed 340 °C. The adhesive diamond interlayer thickness was 180 nm.

The NCD layers were next prepared in a focused MW-PECVD system (Aixtron P6). Unlike the linear antenna MW-PECVD system, high diamond growth rates at higher temperatures are characteristic of what is commonly achieved in the focused MW-PECVD system. In this step, two layers with different thicknesses were prepared. The layer thickness was defined by the deposition time. The diamond layer’s thickness was 230 nm for 3 h of deposition and 720 nm for 8 h. The process parameters were as follows: MW power 2000 W, working pressure 40 mbar of the 300 sccm H_2_ and 3 sccm CH_4_ gas mixture, and the substrate temperature did not exceed 600 °C.

After NCD deposition, it was necessary to functionalize the diamond layer by H-termination in a hydrogen plasma. The hydrogenation was done for 10 min in a pure H_2_ atmosphere under the same MW power, working pressure, temperature, and H_2_ flow conditions as the NCD growth step.

#### 2.1.2 GO-based Layers

GO was prepared using the modified Hummers method^31^ from graphite powder (Merck 99.5% purity, particle size <50 μm) according to the procedure described previously by Sun et al.^37^ This method consisted of several steps. The first step of the synthesis was the initial intercalation of 1 g of powder graphite (Merck, mesh <50 μm) by grinding in an agate mortar with 50 g of sodium chloride (pure for analysis (PA)) for a minimum of 30 min. Then, the mixture was washed several times with warm (∼70 °C) deionized water to completely remove sodium chloride,^31^ filtered on Teflon filters with a pore size of 0.45 μm, dried at room temperature, and transferred to the reaction vessel. In the next step, 23 mL of concentrated sulfuric acid (VI) (96% PA) was added to the graphite and stirred using a magnetic stirrer (∼250 rpm) for 8 h. Then, in an ice bath (temperature of the mixture under 0 °C), 3 g of potassium permanganate (VII) was gradually added until the green color of the suspension appeared. In the next step, the mixture was heated and stirred for 30 min at ∼40 °C and then at ∼70 °C for 45 min. During this time, an increase in the viscosity of the mixture and a change in its color from dark green to brownish were observed. The last step was the addition of 46 mL of deionized water and heating the mixture to ∼100 °C for 30 min. The reaction was terminated by adding 160 mL of deionized water and 10 mL of hydrogen peroxide (30% PA). The resulting mixture was filtered on Teflon filters with a pore size of 0.45 μm and washed several times with a 5% hydrochloric acid solution to remove unreacted Mn^2+^ ions. The reaction product was then washed extensively with deionized water. The final product was flooded with acetone and homogenized using an 18 W ultrasonic horn for 120 min. The GO obtained this way was brown and formed a stable dispersion in acetone. The concentration was 1 g of GO in 25 mL of acetone.

The GO reduction reaction was carried out using ascorbic acid according to the protocol proposed by Zhu et al.^38^ The aqueous suspension of GO was mixed with a 0.1 M aqueous solution of ascorbic acid in a 1:1 volume ratio. The reaction mixtures were then placed in an ultrasonic bath and heated to 60 °C for 60 min. During the reaction, the mixture changed from dark brown to black. After 60 min, an excess of 30% hydrogen peroxide solution was added and kept at 60 °C for another 30 min. The addition of hydrogen peroxide was intended to oxidize the unreacted reducing agent. The reaction product was filtered under a vacuum through filters with a pore size of 0.45 μm. The last step was washing with ethanol and water. The reaction products were stirred in acetone.

The thiol functionalization of GO was carried out according to the protocol described by Bachmatiuk et al.^39^ The first step was to prepare the mixture of 1 g of GO with 500 mL of toluene and 1 g of phosphorus (V) sulfide. The mixture was refluxed (110 °C) for 7 days. The finished product was filtered on Teflon filters with a pore size of 0.45 μm, washed with toluene until the yellow color of the filtrate disappeared, and then washed with deionized water. The samples were stirred in acetone.

**Figure 1:**
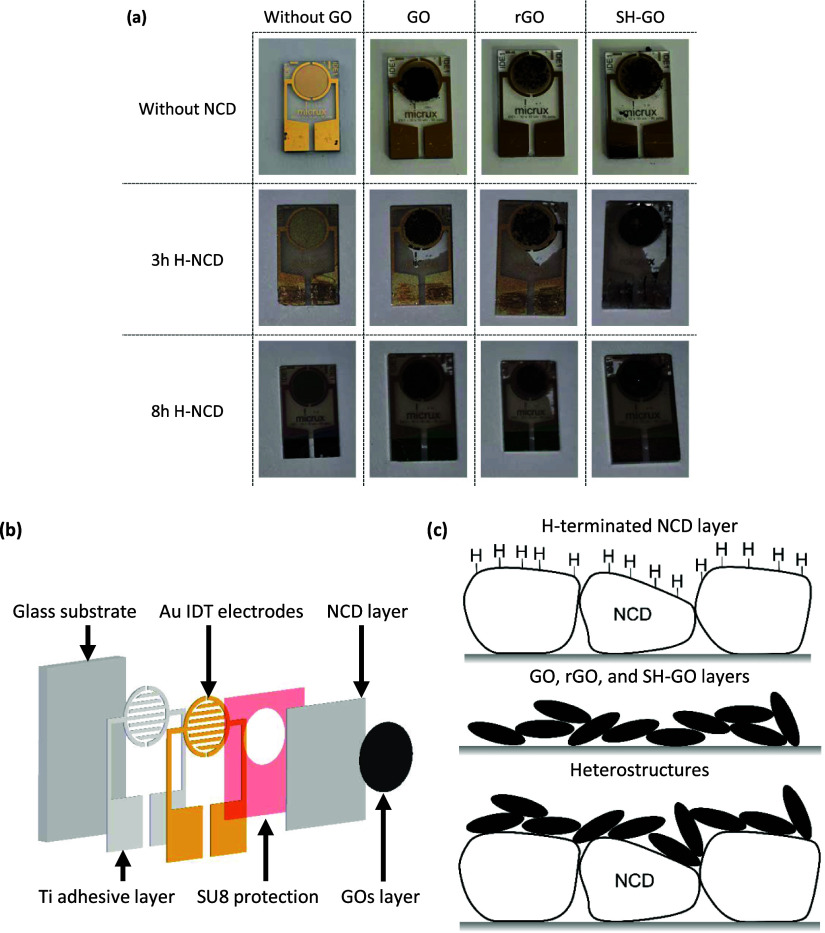
(a) Photographs of the prepared sensors after the sensing experiment, (b) exploded schematic view, and (c) schematic layer cross-section of all fabricated and evaluated sensors.

The GO layer was prepared by using the drop-casting method on the IDT structure (pure gas sensors) and the diamond layer (heterostructures). GOs layers were prepared with a thickness of up to 500 μm in the center of the IDT and spread over 3 mm in diameter. Figure 1 shows photographs and a schematic view of all 12 prepared sensor variants.

### 2.2 Characterization of Sensors

**Figure 2:**
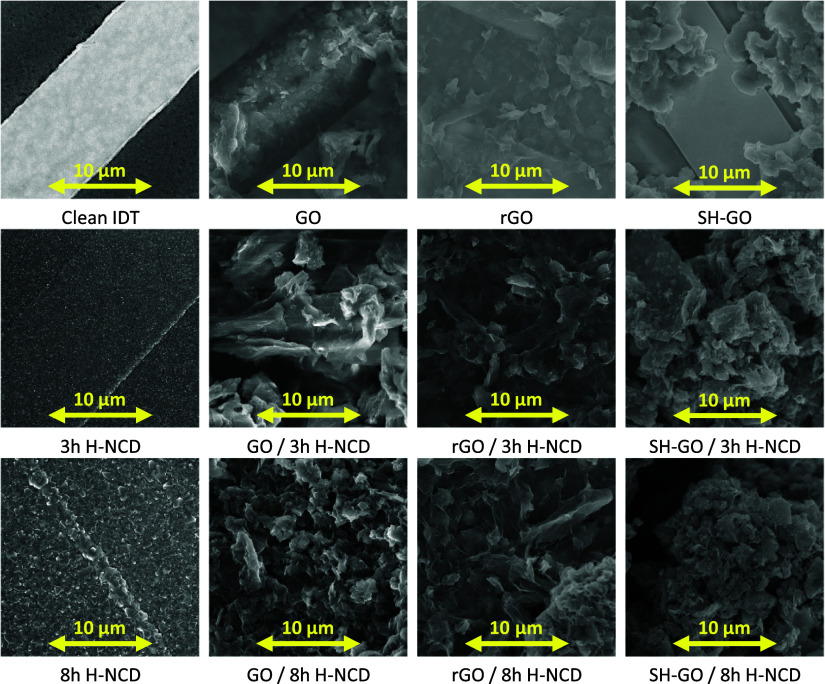
SEM images of all tested sensors.

A scanning electron microscope (SEM) Tescan MAIA 3 measured the surface morphology of the prepared sensors. The SEM image is shown in Figure 2. The fabricated H-NCD film has a homogeneous and continuous surface free of voids over the pad area and glass substrate. The average grain size distribution was 150–300 and 400–700 nm for samples grown for 3 and 8 h, respectively.

**Figure 3:**
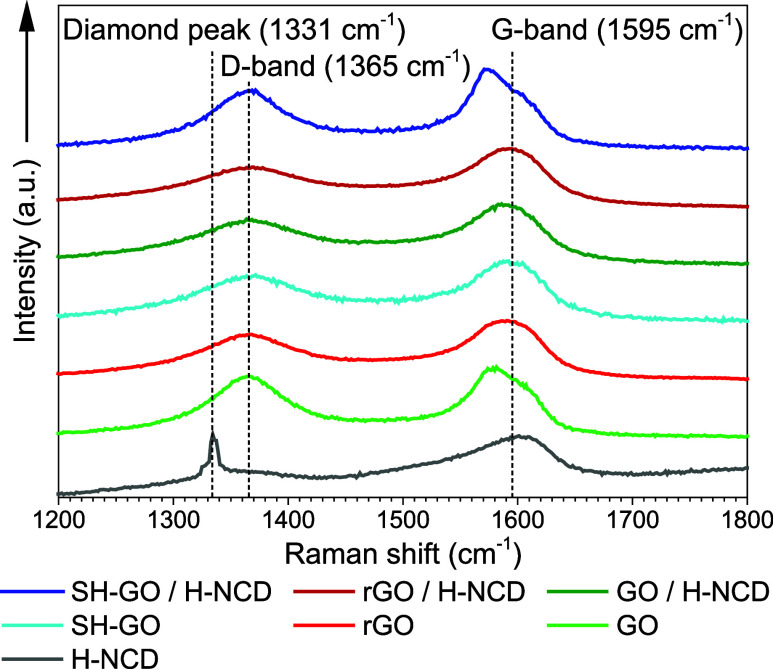
Raman spectroscopy of 8 h H-NCD and GOs-based layers (GO, rGO, and SH-GO) without and with 8 h H-NCD normalized to the G-band.

The GO-based materials created nanoflakes, which enlarged the active area between the gas and sensing materials. The chemical structure was measured with a Renishaw inVia Reflex Confocal Raman microscope with a 442 nm excitation wavelength. Raman spectra of the sensors are illustrated in Figure 3. GO-based materials exhibit typical Raman spectra. In these spectra, two broad bands are labeled as D and G at 1365 and 1595 cm^–1^, recognized as disordered sp^2^ carbon and graphitic phases. 8 h H-NCD exhibits a typical diamond peak at 1331 cm^–1^.

**Figure 4:**
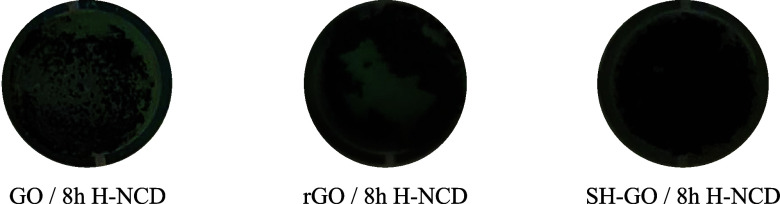
Photographs of GOs sensor active parts 6 months after drop-casting.

The adhesion and stability of the prepared layers are demonstrated in Figure 4. These photos were taken 6 months after the GOs were applied. The photographs show that only the SH-GO/8 h H-NCD sensor achieves good homogeneity. On the other hand, GO/8 h H-NCD and rGO/8 h H-NCD have lower adhesion, and the GO and rGO were peeled off from 8 h H-NCD. Degradation of the layers (peeling off) was reflected by a change in the resistance Δ*R*.

The characterization of individual materials (H-NCD and GO layers) using AFM, XPS, FTIR, and other techniques has already been provided, as described in previously published articles.^29,31,36,40,41^

### 2.3 Measurement Apparatus for Gas Sensor Testing

**Figure 5:**
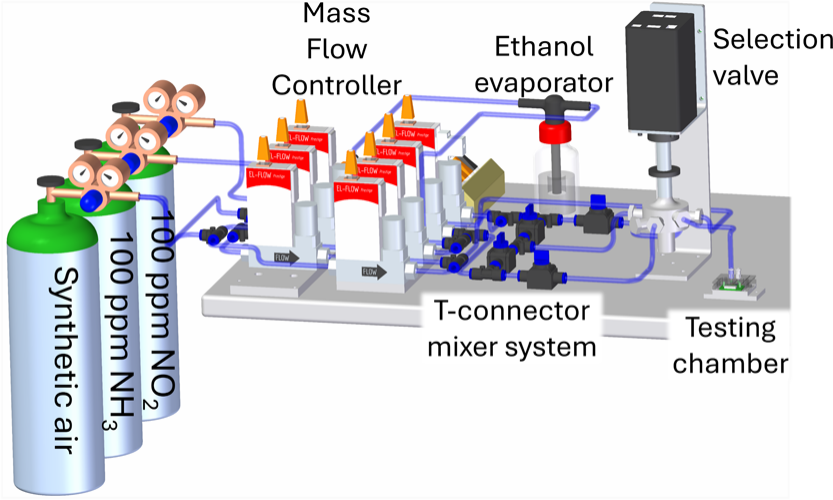
Diagram of the gas sensor response measuring apparatus.

A measurement apparatus (Figure 5) was used to measure the responses of the fabricated sensors. The apparatus comprises 7 mass flow controllers (MFCs), a liquid evaporator, a four-input selection valve, a testing chamber, and a source measure unit (SMU). After the MFCs, a T-connector mixer system uses turbulent mixing of gases and ethanol vapor. The mixtures are selected by a Valco EUTA selection valve with 4 inputs and 2 outputs. The resistance change is measured using an SMU, Keithley SourceMeter 2401, with a four-wire DC resistance measurement (Kelvin resistance measurement). The Kelvin resistance measurement arrangement eliminates the resistance of spring pins, which are used in a custom gas testing chamber specially designed for Micrux substrates with small volumes (about 0.2 cm^3^), enabling fast-changing testing gases. The PT1000 sensor measures the temperature in the chamber, with an average value of 21.5 °C. The prepared sensors were measured with a constant voltage measurement with a nominal value of 0.1 V.

The apparatus is capable of operating with three independent gases (in our case, ethanol vapor, NH_3_, or NO_2_) and a carrier gas in target 1–100 ppm concentrations. Synthetic air (80% N_2_ + 20% O_2_) was used as a carrier and dilution gas. The concentration and flow were set by 7 flow meters. The NH_3_ and NO_2_ were used from a bottle with a manufacturer’s declared 100 ppm concentration. The concentration of ethanol vapor was calculated from the amount of ethanol evaporated in an evaporator and the measured flow of the carrier gas. The commercial sensor (Amphenol VOC-200) verified a calculated amount of ethanol vapor. The direct use of ethanol vapor from the liquid phase is more like real evaporation in industry and the environment.

In all gas sensing experiments, the commercial or fabricated sensors were individually loaded and tested in the test chamber.

### 2.4 Gas Sensor Performance

The responses to three gases (ethanol vapor, NH_3,_ or NO_2_) were measured at room temperature (21.5 ± 0.5 °C). SMU measured the sensors with a constant voltage of 0.1 V using a Kelvin resistance measurement setup to approximate the real application. The sensor response Δ*R* (resistance change) was calculated from eq 1, where *R* represents the actual resistance and *R*_0_ is the steady-state resistance.
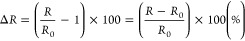
1

## 3 Measurements and Results

**Figure 6:**
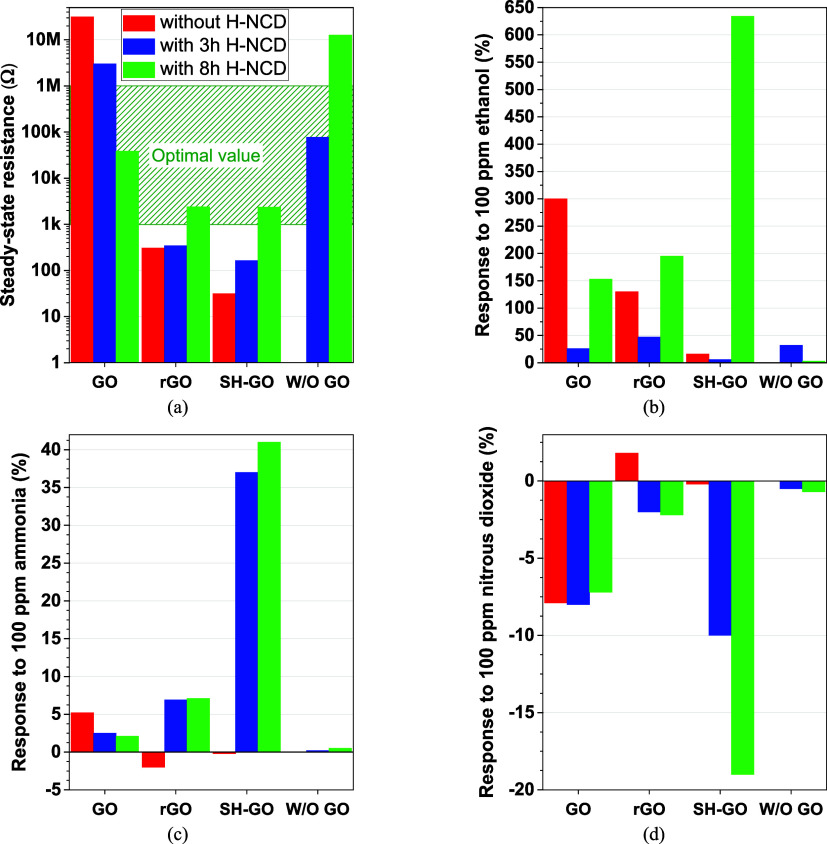
(a) Steady-state resistances *R*_0_ in synthetic air, (b) Δ*R* responses to 100 ppm of ethanol vapor, (c) Δ*R* responses to 100 ppm of ammonia, and (d) Δ*R* responses to 100 ppm of nitrous dioxide of all tested sensors (W/O GO – without any GOs layer).

The first measured parameter was steady-state resistance *R*_0_ in the presence of synthetic air. The obtained values of *R*_0_ are shown in Figure 6a. Next, the sensor responses were measured. The gas-sensitive layers exhibited different Δ*R* resistance changes in the presence of the target gases. Figure 6b–d shows the responses to 100 ppm of gas mixtures at room temperature. This concentration was determined based on the effect on the human organism.^6,7^ A bare IDT structure without surface modifications had no response (i.e., infinite *R*) to any gases.

Figure 6a shows that the optimal value *R*_0_ for low-power devices (from 1 kΩ to 1 MΩ)^2^ was achieved for GO/8 h H-NCD, rGO/8 h H-NCD, SH-GO/8 h H-NCD, and 3 h H-NCD sensors. Higher resistance *R*_0_ would require a higher nominal voltage for a suitable current. A lower resistance *R*_0_, on the other hand, would require a lower nominal voltage under the noise level, which increases the measurement error. From these sensors, the best response of the SH-GO/8 h H-NCD structure achieves a value of 634% for ethanol, 41% for ammonia, and −19% for nitrous dioxide. The pure 8 h H-NCD with higher thickness has a lower response to ethanol than 3h H-NCD, as shown in Figure 6b. The thicker layer has higher resistance *R*_0_ of the bulk material, which has no response to the gases. This observation clarifies that using the thin NCD film with the highest possible roughness is advantageous. The highest roughness increases the adhesion between the two materials and the active surface for the reaction between the gases and the heterostructure.

**Figure 7:**
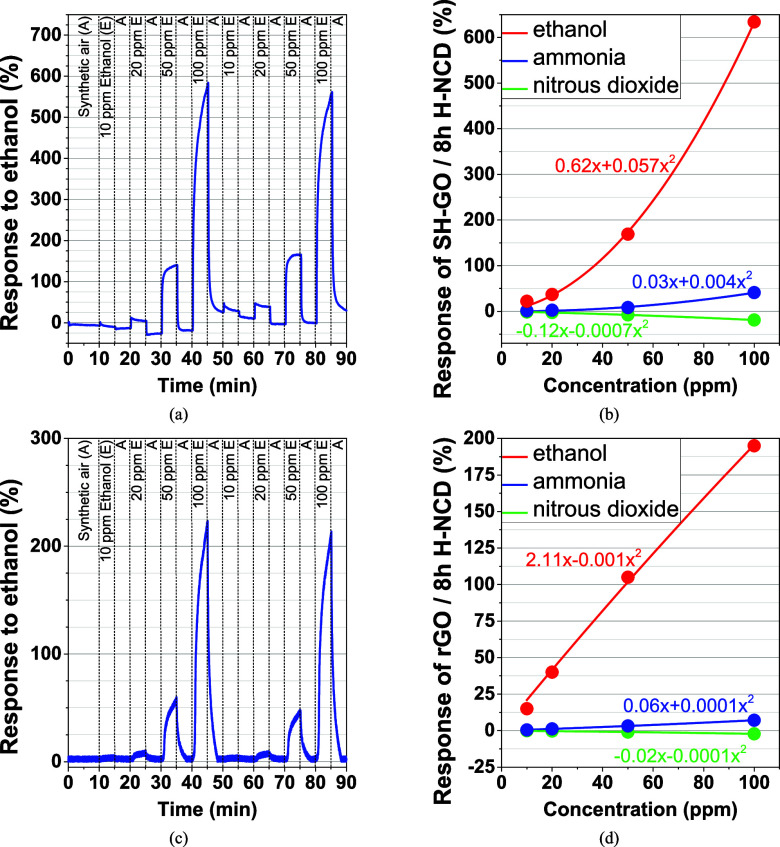
Δ*R* responses to selected concentrations of SH-GO/8 h H-NCD to (a) ethanol vapor and (b) all target gases with polynomial fits; and Δ*R* responses of rGO/8 h H-NCD to (c) ethanol vapor and (d) all target gases with polynomial fits.

The response of SH-GO/8 h H-NCD to various concentrations of ethanol vapor is shown in Figure 7a,c for rGO/8 h H-NCD, respectively. The time responses of other sensors were omitted because they exhibit poor adhesion between layers (Figure 4 for GO/8 h H-NCD), nonoptimal steady-state resistances, and these heterostructures impaired responses to target gases. The dependences of the response on the target gas concentrations are shown in Figures 7b for SH-GO/8 h H-NCD and Figure 7d for rGO/8 h H-NCD. The responses were measured from 10 to 100 ppm, with a response of up to 634%. The ethanol response of SH-GO/8 h H-NCD exhibits a polynomial/exponential-like character to increasing concentration. The calculated theoretical limit of detection (LOD),^42^ 3σ_air_/*R*_0_ (σ_air_ is the standard deviation of resistance in the presence of synthetic air), according to Huang et al.^43^ and Rossi et al.,^44^ is 100 ppb for SH-GO/8 h H-NCD and 5 ppm for rGO/8 h H-NCD. The response/recovery times were calculated as the time between 10 and 90% of the response, respectively, between 90 and 10%.^2,42^ The SH-GO/8 h H-NCD response time calculated for 100 ppm of ethanol vapor was 272 s. The recovery time was 34 s. In comparison, the pure SH-GO material achieves a similar time for response and recovery, with a range of 100–150 s.^31^ The pure 8 h H-NCD achieves a value of about 350 s.^14^

**Figure 8:**
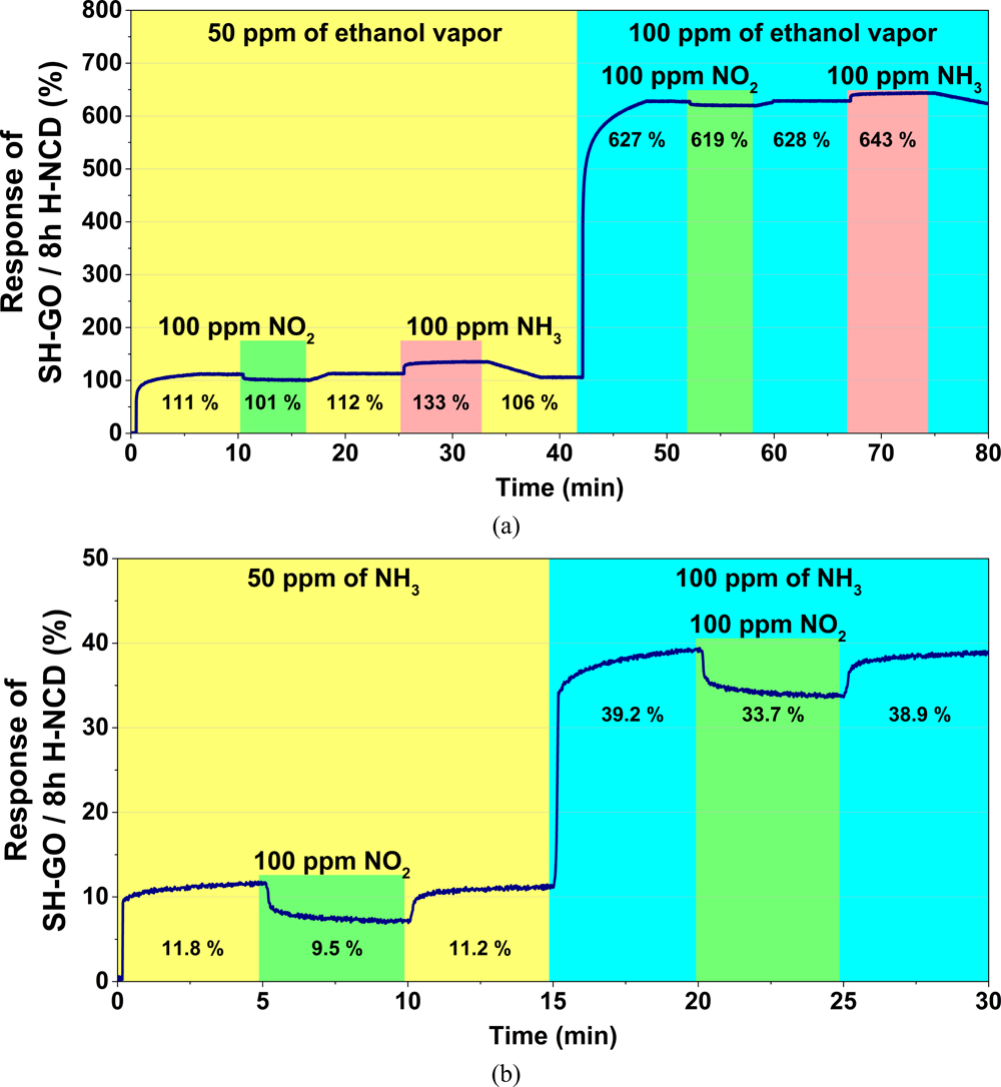
Cross-selectivity responses of SH-GO/8 h H-NCD to (a) two ethanol concentrations (50 and 100 ppm) under the influence of 100 ppm of NO_2_ and 100 ppm of NH_3_ and (b) two NH_3_ concentrations (50 and 100 ppm) under the influence of 100 ppm of NO_2_.

The cross-selectivity response of SH-GO/8 h H-NCD is shown in Figure 8. The first graph (Figure 8a) shows the change in response when another gas (100 ppm of NH_3_ and 100 ppm of NO_2_) is added to the two ethanol vapor concentrations (50 and 100 ppm). The response to pure ethanol vapor is significantly higher than that to other gases, and the influence is almost negligible. In the case of 100 ppm of ethanol vapor, the response is changed in the presence of NO_2_ to only −8%, respectively, and 15% for NH_3_. This change is only −1.3% for NO_2_ and 2.4% for NH_3_ compared to the stable value. The second graph (Figure 8b) illustrates the change in response when 100 ppm of NO_2_ is added to the two concentrations (50 and 100 ppm) of NH_3_. The response change from the stable value for the mixture of 50 ppm of NH_3_ and 100 ppm of NO_2_ is −2.3% and −5.5% for the mixture of 100 ppm of NH_3_ and 100 ppm of NO_2_. In comparison to the stable value, this change is about −30% for 50 ppm of NO_2_ and approximately −14% for 100 ppm of NO_2_.

**Figure 9:**
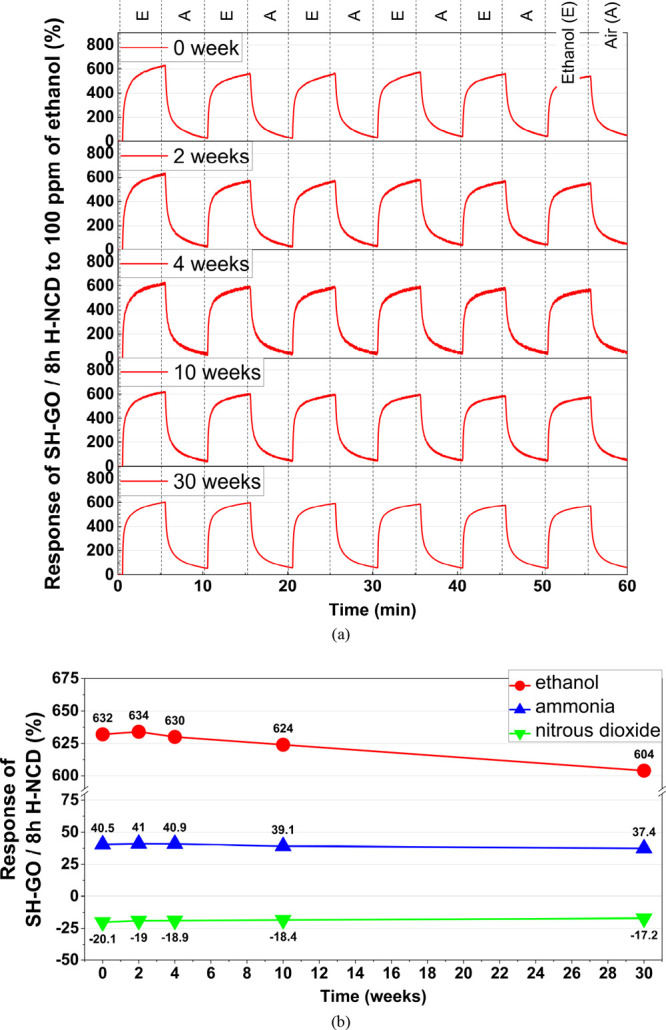
Long-term time dependence of SH-GO/8 h H-NCD response to (a) 100 ppm of ethanol vapor and (b) 100 ppm concentrations of all target gases.

Figure 9 illustrates the long-term stability of SH-GO/8 h H-NCD over a 30-week period after the fabrication of the sensor. Figure 9a depicts the time responses of the sensor to 100 ppm of ethanol vapor, while the responses of all target gases measured at a concentration of 100 ppm are listed in Figure 9b. The sensor response demonstrates a decrease over time. The measured responses show that this sensor exhibits good long-term dependence without any noticeable degradation over time. Only the absolute value of the response decreases. In the case of ethanol vapors, the response decreased from 632 to 604% after 30 weeks (approximately 7 months). This change is approximately 5% from the initial reading. The decreasing response exhibits a linear dependence, thereby allowing for the implementation of a straightforward calculation to correct this error.

## 4 Discussion

The electronic properties and responses of prepared sensors at room temperature are shown in Figures 6 and 7 and summarized in Table 1. Experimental results show that the SH-GO/8 h H-NCD dramatically enhances the responses to ammonia, nitrous dioxide, and ethanol vapor, about 40 times better response to ethanol in comparison with pure SH-GO, respectively 20 times with pure H-NCD (3h H-NCD having better response than 8 h one). Similarly, in the case of gases, the responses are enhanced more than 82 times for ammonia and 27 times for nitrous dioxide. One of the main reasons for improved sensor responses is an increase in the steady-state resistance *R*_0_ from 31 Ω (pure SH-GO) to 2.3 kΩ (SH-GO/8 h H-NCD). Increased resistance is more suitable for conductive sensors because of the lower effect of loading current on the active layer/heterostructure. In the case of GO/8 h H-NCD, the heterostructure exacerbates the sensor responses for pure GO. For rGO/8 h H-NCD, the response to ethanol is slightly improved, but only 1.5 times and 3.5 times for ammonia, respectively. For this reason, the following subsections describe the interaction between the gas molecules and the active layers made from 8 h of H-NCD, SH-GO, and their heterostructure. The gas sensing mechanism of rGO and GO is similar to SH-GO. Humidity can significantly affect the measurement, but in the case of use in industry, membranes can be used to prevent moisture from entering or heating can be used to reduce humidity and improve response at the same time.

**Table 1:**
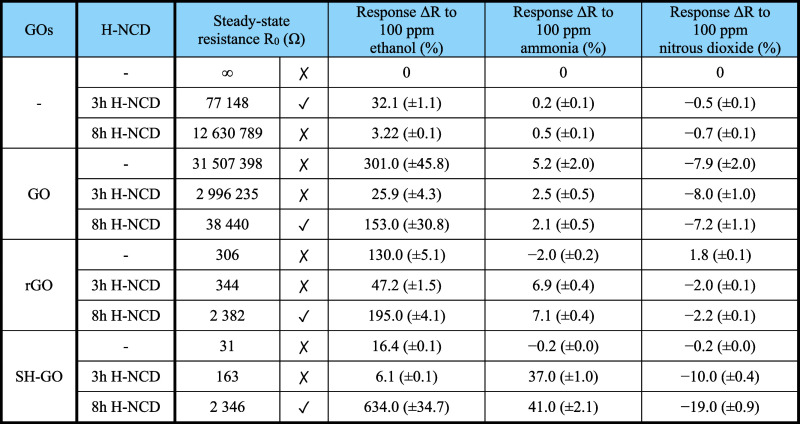
Comparison of Sensor Average Responses from 10 Independent Measurements of Fabricated Sensors (with Standard Deviation σ of the Average Value from 10 Measurements)

Table 2 compares the responses of room temperature gas sensors based on varied materials, heterostructures, and surface modifications. The SH-GO/8 h H-NCD heterostructure achieves perfect responses compared to another sensor in Table 2. Only PANI/CNT^33^ and oxidized SWCNT^15^ respond better than the SH-GO/8 h H-NCD to ammonia and nitrous dioxide, respectively. However, gas sensors for room-temperature detection are mainly based on sp^2^ hybridized carbon nanomaterials with *p*-type semiconductor behaviors. Few strategies to create heterostructures to improve the performance of carbon-based nanomaterial devices/sensors can be distinguished. One is based on nanocomposite formation with conductive metal particles, which improves the sensitivity toward ethanol vapor.^4^ This strategy is based on creating a metal–semiconductor transistor, and they are part of the junction field effect transistor (JFET) group. The subgroup of JFET is PNFET (FET with p–n junction), which is widely explored in literature.^45^ Various materials can be used as semiconductors to create PNFET with carbon nanomaterials, but the most popular are ZnO,^20^ TiO_2_,^16^ SnO_2_,^22^ and MoS_2_.^29,45,46^ Such an approach allows for significantly improved device sensitivity compared to pure carbon structures.

**Table 2:** Comparison of Responses of Various Sensors for Gas Sensing at Room Temperature

gas	gas concentration (ppm)	material system	response (%)	carrier/dilution gas	lit.
ethanol vapor	1000	rGO-Th	0.8	dry air	(23)
	800	SH-RGO	1.3	N_2_	(21)
	700	MoS_2_	27.3	N_2_	(9)
	500	rGO-Th	0.62	dry air	(23)
	100	g-C_3_N_4_/Au	27.3	compressed air	(3)
		g-CN	6	N_2_	(4)
		g-CN/Ag	16	N_2_	(4)
		V_2_O_5_	60	compressed air	(10)
		TiO_2_/Au	16	compressed air	(11)
		ZIF-8/CNT	2	compressed air	(47)
		GO (*similar material as GO in this work*)	20	N_2_	(31)
		AA-PRGO (*similar material as rGO in this work*)	40	N_2_	(31)
		SH-PRGO (*similar material as SH-GO in this work*)	40	N_2_	(31)
		*SH-GO/8 h H-NCD*	634	*synthetic air*	*this work*
	50	TiO_2_/Au	4	compressed air	(11)
		*SH-GO/8 h H-NCD*	169	*synthetic air*	*this work*
	20	*SH-GO/8 h H-NCD*	37	*synthetic air*	
	10	rGO/PPy	3.9	N_2_	(48)
		ANS/rGO	0.2	N_2_	(48)
		*SH-GO/8 h H-NCD*	22	*synthetic air*	*this work*
	5	CNT	1.2	N_2_	(16)
		CNT/TiO_2_	3.6	N_2_	(16)
		rGO ion beam reduction	1.2	compressed air	(17)
NH_3_	100	V_2_O_5_	45	compressed air	(10)
		ZIF-8/CNT	20	compressed air	(47)
		PANI	13.8	compressed air	(18)
		S and N dots/PANI	39.4	compressed air	(18)
		rGO/PANI	10	compressed air	(19)
		H-diamond (*similar material as H-NCD in this work*)	2.5	synthetic air	(14)
		MoS_2_/H-NCD	17.8	synthetic air	(29)
		cpoPcCo/rGO	42.4	N_2_	(48)
		*SH-GO/8 h H-NCD*	41	*synthetic air*	*this work*
	70	PANI/CNT	452	synthetic air	(33)
	50	Oxidized SWCNT	5	N_2_	(15)
		*SH-GO/8 h H-NCD*	8.3	*synthetic air*	*this work*
	20	*SH-GO/8 h H-NCD*	2.4	*synthetic air*	*this work*
	10	*SH-GO/8 h H-NCD*	1.2	*synthetic air*	*this work*
NO_2_	100	H-diamond (*similar material as H-NCD in this work*)	5	synthetic air	(14)
		MoS_2_/H-NCD	15.7	synthetic air	(29)
		*SH-GO/8 h H-NCD*	19	*synthetic air*	*this work*
	50	oxidized SWCNT	25	N_2_	(15)
		*SH-GO/8 h H-NCD*	7.8	*synthetic air*	*this work*
	20	*SH-GO/8 h H-NCD*	2.7	*synthetic air*	*this work*
	10	*SH-GO/8 h H-NCD*	1.5	*synthetic air*	*this work*
	5	rGO ion beam reduction	2.2	compressed air	(17)

Combining two materials with *p*-type semiconductor characteristics allows for transistor-like behavior, significantly improving sensing properties.^19,33^ This type of device is not well-recognized and described in the literature. It should be noted that Wu et al.^33^ reported linear characteristics of polypyrrole/CNT/polyaniline (PPy/CNT/PANI) toward different concentrations of ammonia, while Ding et al.^19^ presented quasi-logarithmic behavior of rGO/PANI sensor toward various concentrations of ammonia and formaldehyde. In contrast, the SH-GO/8 h H-NCD heterostructure presented in this work exhibits slightly polynomial/exponential resistance changes in Δ*R* in ethanol vapor, NO_2_, and NH_3_ at different concentrations. In the presented heterostructure, the combination of highly conductive SH-GO (*R*_0_ of about 300 Ω) with 8 h H-NCD (*R*_0_ of about 10 MΩ) allows to obtain the device with steady-state resistance *R*_0_ of 2 kΩ, in contrary to PPy/CNT/PANI, which has steady-state resistance *R*_0_ of about 30 kΩ.^33^ It suggests that the resistance change Δ*R* toward gaseous media or VOC is specific to the device and depends on the applied combination of materials.

### 4.1 Model of the Gas Interaction Mechanism

#### 4.1.1 H-NCD Layers

**Figure 10:**
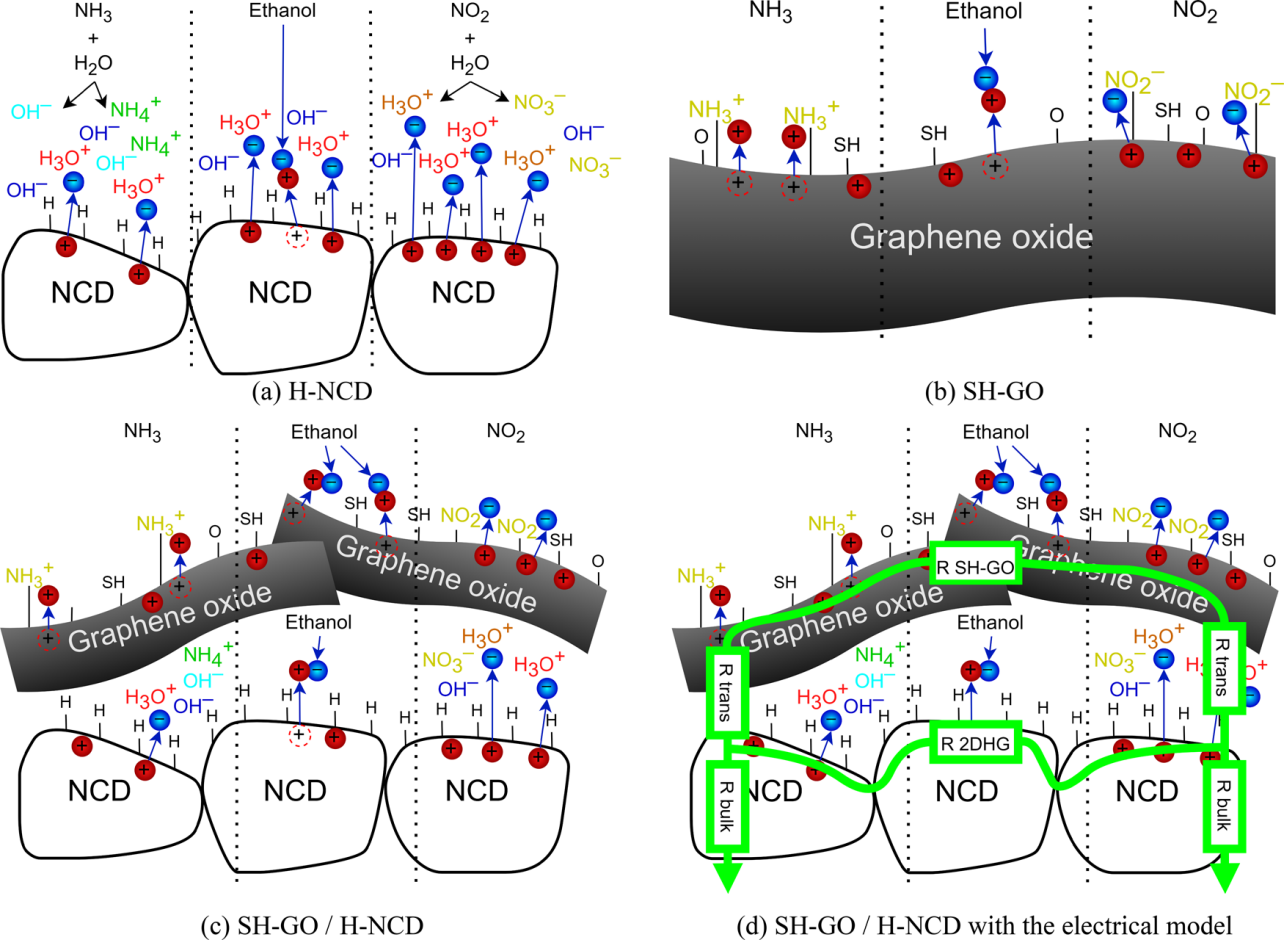
Gas interactions between the gases (NH_3_, ethanol, and NO_2_) and active layers: (a) H-NCD, (b) SH-GO, (c) SH-GO/H-NCD heterostructure, and (d) SH-GO/H-NCD with the electrical model.

A widely established H-terminated diamond surface doping mechanism was used to explain the sensing mechanism. The H-NCD layer reacts with gas molecules via subsurface 2D hole gas, also known as 2DHG.^49^ This subsurface layer is sensitive to the environment, such as humidity, temperature, and organic/inorganic molecules. Previous works have described and explained the gas interaction model between gas molecules and 2DHG.^14,29,30^ Just briefly, in these interactions, water molecules (H_2_O) adsorbed on H-NCD from air humidity dissociate above the diamond surface into H_3_O^+^ and OH^–^ species. It occurs immediately after exposure to air after the CVD deposition or from residual humidity in dry synthetic air. This layer of ions remains on the surface, even when exposed to dry air or other gases. The H_3_O^+^ ions at the H-NCD surface attract electrons from the bulk of the diamond. The attracted electron causes the surplus of holes and induces the *p*-type 2DHG subsurface conducting channel, also known as a “surface transfer doping” process which changes the intrinsic and insulating diamond surfaces to semiconducting ones.^30,41,50,51^ The imbalance of ions changes the number of free charge carriers and thus the resistance. In the presence of reactive gases, the chemical reactions create an imbalance between H_3_O^+^ and OH^–^ ions. In the case of oxidizing gases (NO_2_ in Figure 10a right), the concentration of H_3_O^+^ rises, and the conductivity of 2DHG increases. The process is reversed to reduce gases (NH_3_ in Figure 10a left). The chemical reaction creates OH^–^ ions, and electrons are shifted back to the H-NCD and partially neutralize 2DHG, reducing conductivity. For ethanol vapor, the chemical reaction near the surface creates free electrons,^52^ which partially neutralizes the 2DHG and reduce the conductivity, increasing the resistance (ethanol in Figure 10a center).

#### 4.1.2 GO Layers

Graphene is generally treated as a *p*-type semiconductor without a bandgap, particularly in its single-layer form.^31^ It consists of an interconnected network of aromatic rings. Within each aromatic ring, π electrons are in constant motion, creating a quasi-hole–electron vortex in the material. In the case of GO, which contains various functional groups such as sp^3^ and sp^2^ carbon, hydroxyl, carbonyl, or carboxyl, these modifications interfere with the flow of electricity, effectively disrupting the delocalized π electron–hole tandem and leading to an electrical insulator behavior.^31,38^ rGO is a partially reduced form of graphene oxide with a higher C/O ratio and an improved conductivity pathway.^17^ On the other hand, the SH-GO sample, which incorporates SH groups, is expected to be a versatile material similar to GO with OH groups but with a rebuilt sp^2^ carbon backbone. SH-GO demonstrated excellent conductivity and a notably higher affinity, for instance, toward gold electrodes (than rGO).^31^ In principle, the electron cloud formed by the aromatic rings in the graphene structure can be either enriched with electrons or depleted, resulting in electron enrichment or depletion. Ethanol vapor (Figure 10b center), or reducing gas NH_3_ (Figure 10b left), “shares” its electrons with the electron cloud and reduces the number of holes. Conversely, oxidizing gases like NO_2_ (Figure 10b right) “withdraw” electrons. Notably, the dominant phenomenon is the physical sorption of NO_2_ molecules on the graphene structure, which leads to short-range physical interactions between the electron cloud of the graphene structure and the gases. The primary role of functionalizing graphene-based materials is to enhance selectivity and affinity for specific gases.^31^

#### 4.1.3 SH-GO/H-NCD Heterostructures

This section focuses on the SH-GO/H-NCD heterostructure because this structure achieves the highest sensor response. This structure combines previously described individual gas interaction models, enhancing its sensitivity. It provides a unique material platform where both materials exhibit similar electrical responses to reducing and oxidizing gases (Δ*R*) despite operating on different interaction models with gases. Figure 10c illustrates both materials within the heterostructure, and Figure 10d shows an electrical model connecting them. The gas interaction is influenced by several factors, including surface charge carrier injection, short circuits between H-NCD and SH-GO surfaces, and modulation of diamond subsurface conductivity (close to a transistor effect). While all mechanisms are still under investigation, the sensitive transition/interlayer between materials is likely the primary reason for the heightened response to gases.

Near the H-NCD, water molecules dissociate into OH^–^ and H_3_O^+^, with electrons transferring from the diamond surface. This process induces a 2DHG (two-dimensional hole gas) subsurface layer with reduced hole density (high resistivity around 13 MΩ) in the diamond subsurface (Figure 10a). This layer of ions remains on the surface even when exposed to dry air or other gases. The SH-GO layer exhibits *p*-type conductivity^31,53−55^ due to the partially regained highly organized conjugated structure of aromatic rings characteristic of graphene (low resistance *R*_0_ around 31 Ω) (Figure 10b). Upon joining both materials, a thin film of OH^–^ and H_3_O^+^ molecules remains between layers. The negatively charged OH^–^ then acts as electron donors for the graphene structure, enriching the electron cloud and forming a negatively charged SH-GO layer, thereby increasing the steady-state resistance of *R*_0_. When negatively charged graphene serves as the active layer of the sensor and interacts with NO_2_ (Figure 10c right), the resistance R drops notably due to graphene’s increased electron removal tendency, with NO_2_ acting as the electron acceptor. In the case of ethanol (Figure 10c center) and the reducing gas NH_3_ (Figure 10c left), the gases enrich the electron cloud of the aromatic system simultaneously with the H-NCD bottom layer, leading to an enhanced resistive response of the hybrid sensor. In the case of multiple gases, the resulting response is determined by the ratio of OH^–^ and H_3_O^+^ molecules and the concentration of the free charge carriers in 2DHG. Furthermore, this combination creates the most sensitive part of the sensors: the transition interlayer region between layers. This region exhibits sensitive nonlinear characteristics in response to the gas presence and changes in free charge carrier concentrations in both layers. Changing the concentration of free charge carriers in one layer (SH-GO or H-NCD) influences the concentration in the other layer, thus changing the transient resistance between the two layers. This theory finds support in experiments with SH-GO/O-NCD (oxygen-terminated NCD), where the absence of a 2DHG and free charge carriers results in a bulk resistance *R*_bulk_ similar to H-NCD (>100 MΩ) and negligible response to gases. Thus, the presence of the *p*-type subsurface layer (2DHG) is crucial for establishing a sensitive transition between both layers.

**Figure 11:**
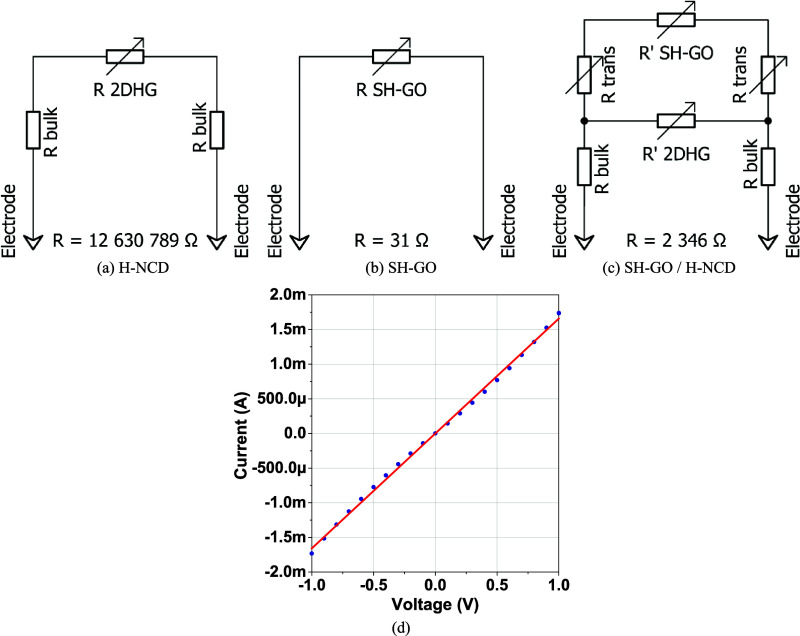
Electrical model representations of active layers: (a) H-NCD, (b) SH-GO, (c) SH-GO/H-NCD heterostructure, and (d) current–voltage characteristic (*I*–*V* curve) of SH-GO/8 h H-NCD.

Simple electro-mathematical simulations of sensors in the Maple and Wolfram Alfa programs supported this phenomenon. Electrical models of each active layer are shown in Figure 11. A sensitive 2DHG layer and a nonsensitive bulk resistance *R*_bulk_ can replace the H-NCD (Figure 11a). The SH-GO (Figure 11b) is the only simple resistance *R*_SH-GO_ of the sheet. The heterostructure (Figure 11c) parallels these two diagrams with the transition resistance *R*_trans_ between H-NCD and SH-GO. Figure 11 d illustrates the *I*–*V* curve of SH-GO/8 h H-NCD, which demonstrates a linear response to increasing current and voltage (in the range of −1 to 1 V). Hence the diode effect between the electrodes and resistive *R*_bulk_ for a constant voltage of 0.1 V can be excluded.

The simulation works with a variation of *R*_2DHG_, *R*_trans_, *R*_bulk_, and R_SH-GO_ to achieve the highest possible response. The simulations show that the best values in the heterostructure are 2.3 kΩ toward the serial combination of *R*_SH-GO_ and *R*_trans_, 1252.6 kΩ toward R_2DHG_ in H-NCD, and 0.45 kΩ toward *R*_bulk_ of H-NCD. The *R*_trans_ is likely more significant than SH-GO. The resistance ratio is estimated to be thousands of Ohms to the *R*_trans_ and tens to hundreds of Ohms to the *R*_SH-GO_. This combination achieves a response of 493.65% for ethanol. The simulated response value is the closest to the measured response Δ*R* from all of the simulations, but the measured value is still higher. For this reason, changing the resistance cannot be the only reason for improving the response. It can also be concluded that this structure exhibits a slightly transistor-like mechanism. In the presence of gases, SH-GO changes the number of holes and conductivity. A change of free charge carriers may change the potential and probably displacement of H_3_O^+^ and OH^–^ molecules. These molecules cause the imbalance between OH^–^ and H_3_O^+^ near the H-NCD and thus the concentration of holes in 2DHG.

## 5 Conclusions

Conductive gas sensors based on carbon structures were fabricated and successfully evaluated against oxidizing and reducing gases (NO_2_ and NH_3_, respectively) and ethanol vapor in the 10–100 ppm range at room temperature (21.5 ± 0.5 °C). Good sensitivity was achieved by proper selection of the active/sensing layer. GO, rGO, SH-GO, and their heterostructures with H-NCD were studied. SEM and Raman spectroscopy also analyzed the active layers to verify the morphology and chemical structure of pure materials and their heterostructures.

Pure H-NCD and GOs showed poor to satisfactory responses regarding gas sensing properties at room temperature. However, combining them into a heterostructure significantly improved the gas sensing parameters of NH_3_ and NO_2_. The best sensitivity was achieved for the combination of SH-GO and 8 h H-NCD. Such heterostructure enhanced the response 40 times for ethanol vapor, 82 times for NH_3_, and 27 times for NO_2_ compared to pure materials. The results are promising for detecting various gases, including those used (NO_2_, NH_3_, and C_2_H_5_OH). The theoretical LOD is 100 ppb for SH-GO/8 h H-NCD and 5 ppm for rGO/8 h H-NCD. The SH-GO/8 h H-NCD response time for 100 ppm ethanol vapor was 272 s, and the recovery time was 34 s. In comparison, the pure SH-GO material achieves a similar time for response and recovery with a range of 100–150 s.^31^ The pure H-NCD achieves a value of about 350 s.^14^ The cross-selectivity measurements indicate that the SH-GO/8 h H-NCD exhibits relatively high selectivity for ethanol vapor. The change is only −1.3% for NO_2_ and 2.4% for NH_3_ compared to the stable value for the combination of 100 ppm ethanol and 100 ppm other gas. With regard to the selectivity between NH_3_ and NO_2_, the sensor displays a greater degree of selectivity for NH_3_, albeit with a considerably smaller size. This change is approximately −14% for 100 ppm of NO_2_ in comparison to the NH_3_ stable value. The SH-GO/8 h H-NCD heterostructure also shows suitable temporal parameters, as after 6 months, the structure is visually unchanged (no peeling off), and the results are comparable. The measured responses of the sensor demonstrate that it exhibits good long-term stability without any noticeable degradation in its time-dependent performance over an extended period. The only observed change was a slight decrease in the absolute value of the sensor’s response. In the case of ethanol vapor detection, the sensor’s response decreased from an initial value of 632 to 604% after 30 weeks (approximately 7 months). This change represents approximately a 5% reduction from the initial value. Notably, the decreasing response exhibits a linear dependence, which allows for the implementation of a straightforward calculation to correct this minor error.

Finally, this article presents the interaction model explaining the detection mechanism for the SH-GO/8 h H-NCD heterostructure. The formed heterostructure comprising *p*-type materials created a synergistic effect that enhanced the sensor response. The most important part of the heterostructure is the transition interlayer between the materials, which is mostly affected by the presence of the target. This part is sensitive to nonlinear characteristics of the target presence and the change in free charge carriers’ concentration in both layers. A change in the concentration of free charge carriers in one layer has a marked effect on the concentration in the other, thereby modifying the transient resistance between the two layers. Both simple electro-mathematical simulation and experiments with SH-GO/O-NCD support this model. As demonstrated by the experiments, the absence of a 2DHG and free charge carriers results in a negligible response to gases.

In conclusion, the main advantages of this new structure are its small size, active part diameter of only 3.5 mm, room temperature functionality, excellent response to ethanol vapor, and reasonable response to evaluated and industrially essential gases. These findings indicate that the sensor developed in this work is capable of maintaining its performance characteristics over the long term, making it a promising candidate for real-world applications that require reliable and stable gas detection capabilities. The linear nature of the response degradation further simplifies the process of compensating for this minor change, ensuring the sensor’s continued accuracy and reliability in prolonged use. It is important to note that the humidity and other environmental factors can markedly influence the measurement. However, in the context of industrial applications, the utilization of membranes can effectively prevent humidity from the environment, while heating can stabilize the temperature, reduce humidity, and enhance the response. Future investigations will concentrate on testing responses in the presence of moisture and combinations of diverse gases in the mixture to ascertain the cross-selectivity and other industrially essential gases with varying concentrations.

## Data Availability

The open data are available at: https://zenodo.org/records/12703965, doi: 10.5281/zenodo.12703965.
